# Rapid gas–liquid reaction in flow. Continuous synthesis and production of cyclohexene oxide

**DOI:** 10.3762/bjoc.18.67

**Published:** 2022-06-13

**Authors:** Kyoko Mandai, Tetsuya Yamamoto, Hiroki Mandai, Aiichiro Nagaki

**Affiliations:** 1 Department of Synthetic Chemistry and Biological Chemistry, Graduate School of Engineering, Kyoto University, Nishikyo-Ku, Kyoto, 615-8510, Japan; 2 Department of Pharmaceutical Science, Faculty of Pharmaceutical Science, Gifu University of Medical Science, Nijigaoka, Kani-city, Gifu Prefecture, 509-0293, Japan

**Keywords:** air, continuous flow, cyclohexene oxide, flow epoxidation, rapid gas–liquid reaction

## Abstract

The enhanced reaction rate in the epoxidation of cyclohexene with air as an oxidant was discovered without any added catalyst utilizing a continuous flow reactor constructed with readily available stainless steel parts and devices. This continuous-flow process demonstrates a significant improvement in reaction time for highly selective epoxide production over the batch process due to the efficient mass transfer between the liquid phase and air. The flow process discovered was operated continuously with good operational stability, evaluated by a constant high yield of cyclohexene oxide, to obtain the desired product with high productivity.

## Introduction

From the past to the present, organic synthesis has contributed to the development of science and technology. With the rapid advances in the 21^st^ century, increasing demand for organic synthesis has led to a strong need for faster and more sophisticated methods.

The gas–liquid reaction is one of the organic synthetic methods of importance because gases are one of the promising reagents concerning environmental aspects. Demands for the use of gases for constructing various functional organic materials should be increased if a reliable and powerful technique for gas-liquid reaction is established. In a conventional batch process, a faster reaction with good efficiency should be pursued either by pressurizing the reaction system filled with gas using an autoclave or by introducing gas into the reaction mixture with bubbling [1]. Thinking about the implementation of gas–liquid reactions, especially at the industrial manufacturing level, the conventional batch technique has irreconcilable limitations.

Cyclohexene oxide is one of the key starting materials for manufacturing various functional organic compounds and materials such as polymers and chiral organic compounds with cyclohexane moiety [2–6]. Thus, an efficient and fast synthetic method for the production of cyclohexene oxide is highly desired to be developed, taking sustainability for the environment and our society into consideration. The general synthetic procedure for cyclohexene oxide is the epoxidation of cyclohexene [7–8]. Among various oxidizing agents used in the oxidation, a combination of molecular oxygen and aldehydes as a sacrificial agent has been widely studied [9]. However, in general, such a reaction in batch is slow due to the difficulties of performing a gas–liquid reaction in a batch reactor [10]. In addition, even valuable catalysts could not accelerate the reaction with good efficiency [11–12].

**Scheme 1:**
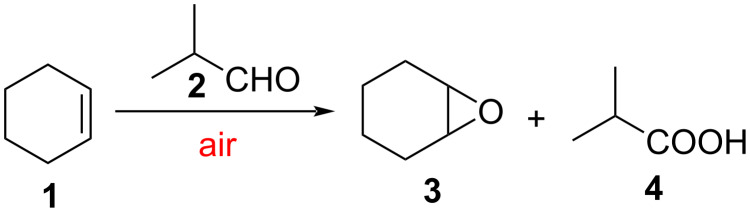
Synthesis of cyclochexene oxide via epoxidation with air in the presence of isobutyraldehyde.

The continuous flow technology has brought a dramatic change and new aspects in organic synthesis [13–23] and has been noticed to provide significant improvement in gas–liquid reactions [24–25]. Thus, we envisioned that such a technology should make the gas–liquid-type epoxidation of cyclohexene using air as a green gaseous reagent faster with good efficiency to synthesize important organic compounds. Previous reports on reactions in a flow system using air as a green reactant [26–27] and epoxidations in a flow system using oxidants other than air [28–30] encouraged us to develop a highly productive flow system with air. Herein, we report that rapid gas–liquid oxidation of cyclohexene with air in the presence of isobutyraldehyde as a sacrificial agent to synthesize cyclohexene oxide is successfully achieved by using a flow technique (Scheme 1). Cyclohexene oxide was selectively produced with high yield in our flow oxidation system using air and within only 1.4 min. The fast epoxidation of cyclohexene without added catalyst in the solution was achieved since the solution of cyclohexene and aldehyde in 1,2-dichloroethane and air could react efficiently inside a pressurized microfluidic channel at high temperature in the flow microreactor. It is important to be noted that precise control of reaction temperature and residence time in our flow system are keys to inhibit overreactions in cyclohexene oxidation and decomposition of oxidants generated from air and aldehyde. Furthermore, the fast epoxidation is applicable for the continuous production process of cyclohexene oxide for 1 hour maintaining stable operation.

## Results and Discussion

### Batch experiment of epoxidation of cyclohexene with air

**Figure 1:**
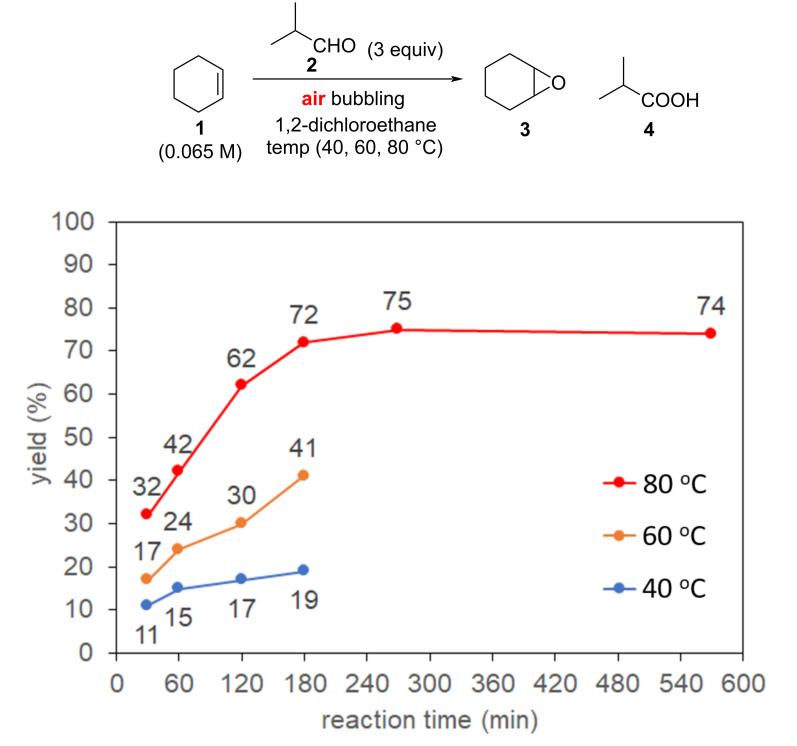
Epoxidation of cyclohexene with air bubbling in batch at various temperature.

As an initial study, we carried out the oxidation of cyclohexene with air using a typical batch-type apparatus. The similar reaction conditions described in the reference were followed to compare the epoxidation with air to that with oxygen [10]. 3 equivalents of isobutyraldehyde were used and the concentration of cyclohexene in 1,2-dichloroethane was set to 0.065 M (see the reaction scheme in Figure 1). The air was introduced into the reaction mixture by bubbling from an air cylinder under atmospheric pressure. The reaction was investigated at different reaction temperatures of 40, 60, and 80 °C (Figure 1). The experiment was carefully carried out using a pear-shaped flask equipped with a reflux condenser cooled to −15 °C to avoid the loss of relatively volatile organic compounds in the reaction mixture. In the reaction at 40 °C, the reaction was very slow to yield the product in less than 20% yield within 270 min. This indicates that the cyclohexene oxidation with air can be hardly promoted at the temperature although the same reaction using bubbling of oxygen yielded cyclohexene oxide in 84% yield (GC) at 40 °C for 270 min [10]. When the reaction was performed with air at 60 °C, a significant increase in yield of the epoxide was observed to reach about 75% after 270 min but no more improvement was achieved. Then, the reaction was carried out at an elevated temperature of 80 °C, resulting in a decreased yield of the epoxide within 270 min as compared to the reaction at 60 °C. This result stemmed from the lowered solubility of air in a solvent at a high temperature to produce peracid from the reaction of aldehyde and oxygen insufficiently, and the epoxidation was deaccelerated. The experimental results revealed that aerobic epoxidation of cyclohexene in a batch reactor required a longer reaction time than 3 h to reach the maximum yield but a moderate yield of the epoxide.

The reaction under pressure requires an extensive pressurizing reactor such as an autoclave. Safety issues, however, would be unavoidable. Introducing gas as a bubble in the reaction mixture is a well-used method to increase the interfacial contact area [1]. However, thinking about the implementation of gas–liquid reactions at the industrial manufacturing level, the conventional batch technique has irreconcilable limitations.

Consequently, the batch operation has definite limitations to meet the demand to establish a highly productive process for industrial manufacturing.

### Investigation of epoxidation of cyclohexene with air in continuous flow system

The flow system for the cyclohexene epoxidation with air was constructed and an investigation of flow conditions, temperature, and residence time was conducted. Subsequently, continuous production of cyclohexene oxide and further investigation to enhance the productivity in the flow system were conducted as described below.

#### Experimental setup

**Figure 2:**
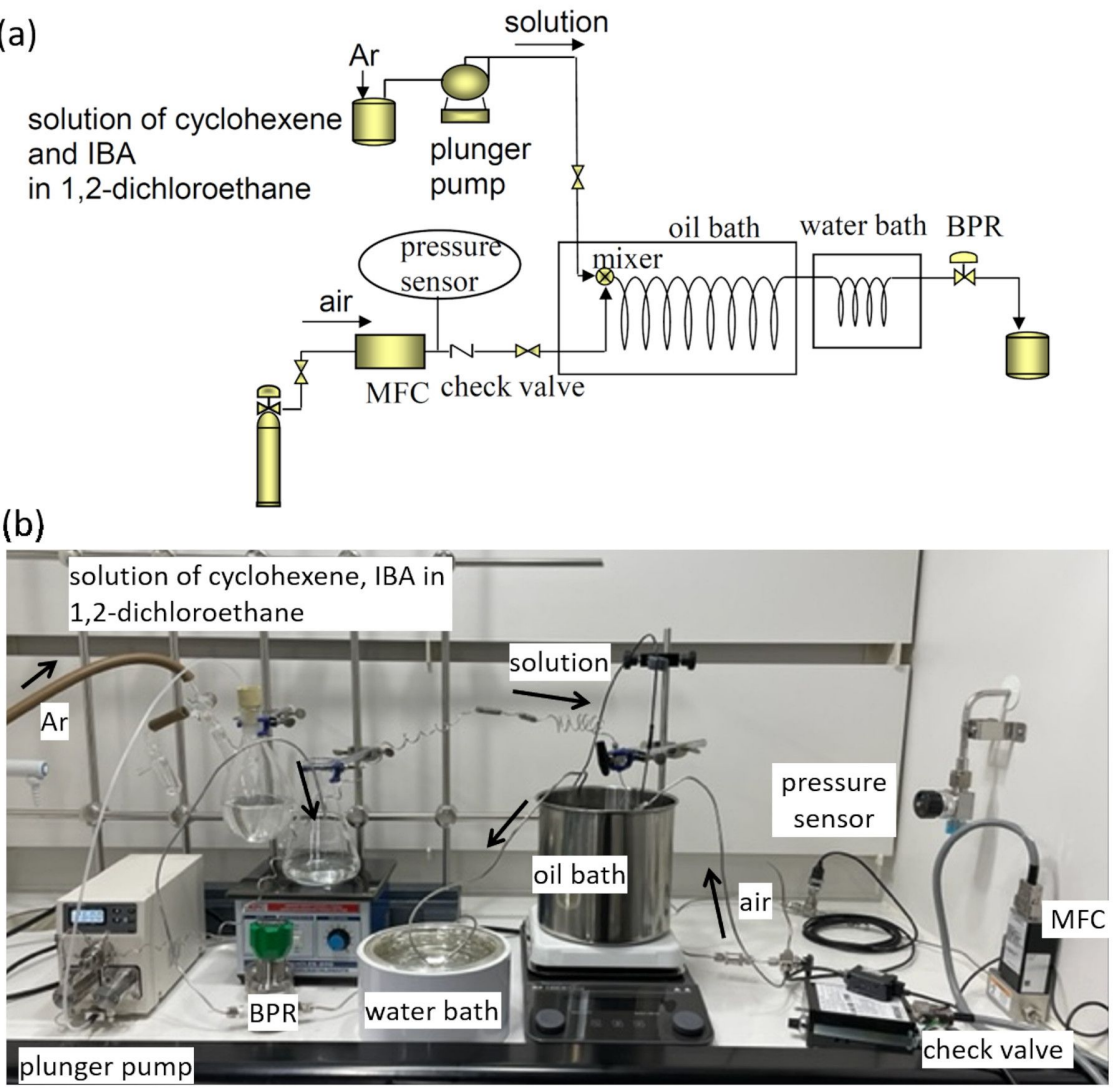
Schematic diagram (a) and photo (b) of the flow reactor used for cyclohexene epoxidation with air. IBA = isobutyraldehyde.

As shown in Figure 2, the system was constructed using commercially available stainless-steel tubing, mixer, joint parts, and devices. For a reactor, widely-used 1/8 inch stainless-steel tubing with a relatively large inner diameter (2.17 mm) was used to establish a readily applicable flow system for industrial use. A solution of cyclohexene and isobutyraldehyde in 1,2-dichloroethane was pumped out by a diaphragm pump. The concentration of cyclohexene in 1,2-dichloroethane was set to 0.065 M for the initial study and three equivalents of isobutyraldehyde were dissolved in the solution. While the solution was sent to the flow reactor at the flow rate of 2.6 mL/min, the air was at the flow rate of 480 mL/min with keeping the molar ratio of aldehyde and oxygen to 1:9. The liquid and gas phases were combined at a T-shaped mixer with a 1 mm inner diameter and flowed through a stainless-steel tube reactor immersed in heated silicon oil for reaction temperature control. The inner pressure was maintained at 0.9 MPa using a back pressure regulator (BPR). The reaction solution was cooled to ambient temperature in a water bath at the position right before BPR and collected in a sample vial. The solution was then immediately diluted with deuterated chloroform for ^1^H NMR analysis.

#### Investigation of the temperature effect

**Figure 3:**
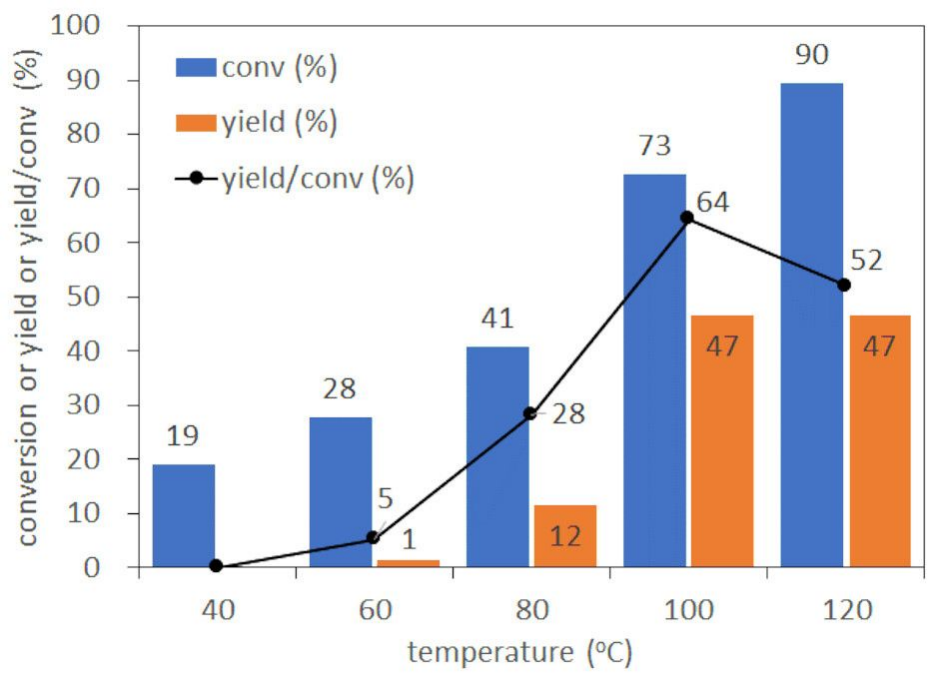
Investigation of reaction temperature in flow epoxidation of cyclohexene at residence time of 0.35 min.

Initially, we set out our research by investigating the effect of temperature on the epoxidation reaction at a residence time of 0.35 min (Figure 3). The temperature range tested was from 40 °C to 120 °C at intervals of 20 °C. At 40 °C, no epoxide was produced in spite of about 20% conversion of cyclohexene. When the temperature was elevated from 40 to 100 °C in increments of 20 °C, conversion and yield increased up to 73% and 47%, respectively at 100 °C. The yield was, however, no more improved from 47% at 100 °C by elevating the temperature to 120 °C even with the highest conversion of 90% attained, indicating the undesired reaction could take place to reduce the epoxide output at 120 °C. The efficiency of the reaction towards the desired epoxidation was clearly demonstrated by calculating the ratio of yield over conversion as black dots in Figure 3. As a result, the highest yield/conversion ratio for epoxidation was obtained in the reaction at 100 °C which is the temperature of choice for the following optimization of the flow epoxidation.

#### Investigation of the residence time

**Figure 4:**
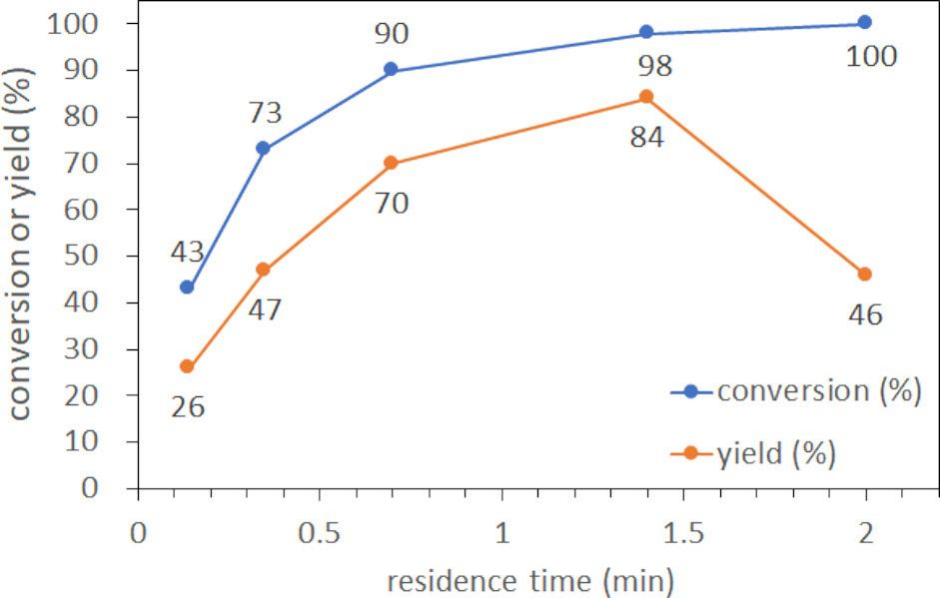
Investigation of residence time in flow epoxidation of cyclohexene at 100 °C.

The residence time of the flow epoxidation was examined at 100 °C from 0.35 to 2 min (Figure 4). Both conversion of cyclohexene and yield of cyclohexene oxide increased as residence time elongated from 0.35 to 1.4 min. However, a drastic decrease in both values was observed at a residence time of 2 min. This result indicates that the epoxide was generated efficiently at a proper residence time, which could be explored readily by performing the reaction in a continuous flow system. To the best of our knowledge, this is for the first time to achieve the epoxidation of cyclohexene using air within only 1.4 min by carefully avoiding any unwanted release which could result in a hazardous situation. In our flow conditions, the volume of air is remarkably higher than that of the solution (480 mL/min of air vs 2.6 mL/min of solution). Although we have no clear evidence for now, the solution could form a very thin layer due to plug-type flow. In such a case, the interfacial area is greatly enlarged in the flow reactor due to the high volume-to-surface ratio. Thus, very efficient contact between reactants in solution and air was achieved to enhance the reaction remarkably. Furthermore, the volume and inner pressure of the reactor were readily and precisely controlled even at the elevated reaction temperature. Consequently, the operation of the gas–liquid oxidation even with air in the flow reactor definitely paved the way for a green and fast oxidation process.

**Scheme 2:**
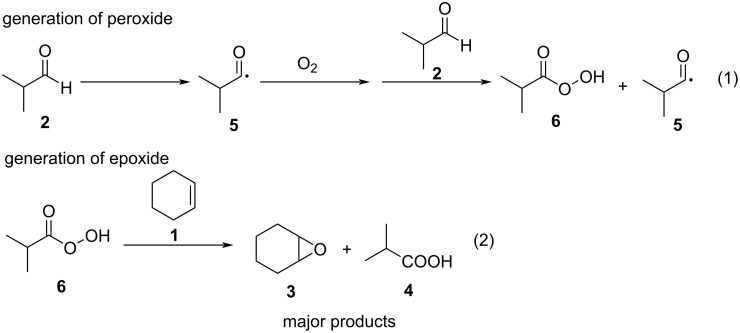
Plausible reaction pathway of the epoxidation of cyclohexene with air in the flow system.

Based on the mechanisms reported in the literature [11,31], we hypothesized the plausible reaction mechanism in our flow system as shown in Scheme 2. Firstly, the peracid **6** was generated via the autoxidation process of aldehyde (reaction 1), which rapidly oxidized coexisting cyclohexene to produce cyclohexene oxide as a major product along with isobutyric acid **4** (reaction 2). Although we did not quantify whether the polymerization of epoxide **3** might proceed when the residence time was elongated, leading to a significant decrease in the yield of cyclohexene oxide (Supporting Information File 1, Figure S2) [32]. Overall, we assume that, in our flow system, the highly efficient contact of acyl radical **5** with oxygen during the autoxidation of aldehyde could produce the peracid **6** very efficiently, which immediately reacts with cyclohexene to generate cyclohexene oxide selectively. This reaction process might take place smoothly and selectively in the microfluidic channel to achieve a high reaction rate.

#### Verification of continuous production

**Figure 5:**
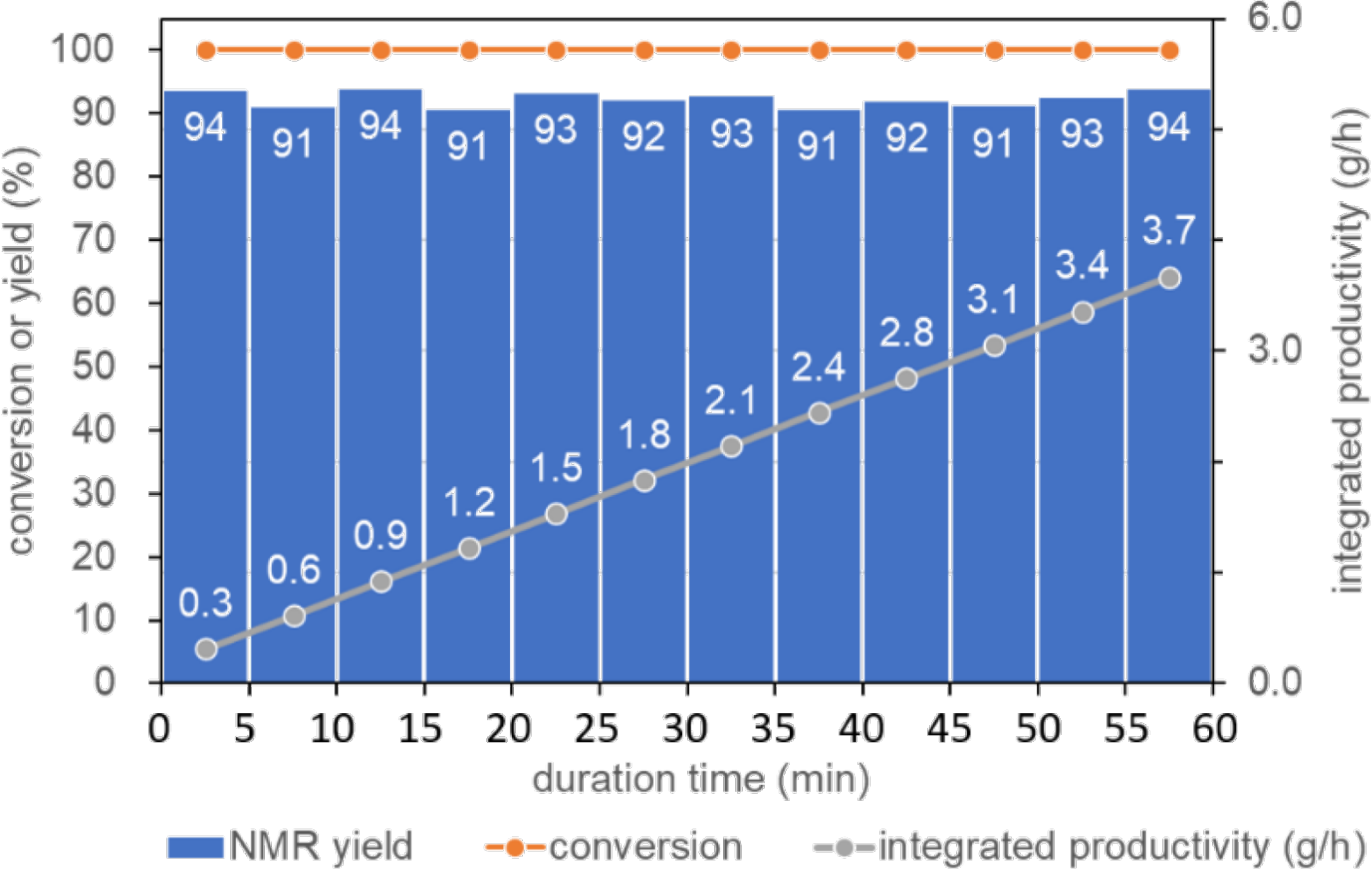
Continuous production of cyclohexene oxide.

The above-mentioned exploitation of optimal conditions for flow cyclohexene epoxidation revealed that continuous flow operation of the reaction provides cyclohexene oxide very fast in the highest yield with the best efficiency using green, inexpensive air. Next, we verified the continuous production of the epoxide with the aim of industrial implementation of this synthetic process (Figure 5). The stability of the continuous flow operation was confirmed during the 1-hour operation as follows. The reaction solution flowing out of the exit was collected every 5 min and analyzed immediately by ^1^H NMR. Yields and conversions were plotted against the operation time as shown in Figure 5. As demonstrated clearly, the production of cyclohexene oxide was maintained constant and high during 1-hour operation. As a result, the productivity was integrated to reach 3.7 g/h reliably, determined by ^1^H NMR analysis.

#### Investigation of the equivalents of isobutyraldehyde and concentration of cyclohexene for further enhancement of cyclohexene productivity

**Figure 6:**
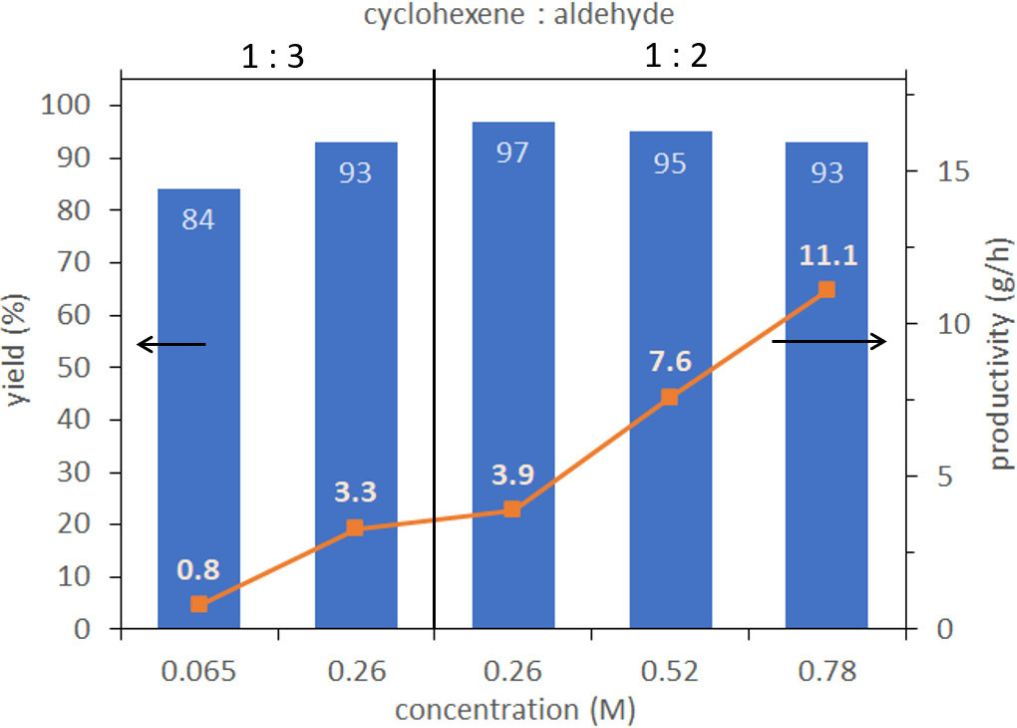
Effect of concentration of cyclohexene and eqivalent of aldehyde.

We turned our attention to enhancing the productivity further. It is significantly important for the industrial operation to increase the productivity by reducing the volume of reagents and organic solvents used for safe, low-cost, and environmentally benign operations. Initially, we decided to maintain the key flow conditions such as the flow rate since it severely affects the fluidic interaction inside a flow reactor and thus the reaction efficiency of this type of gas–liquid reaction. Thus, the flow rate of the solution containing cyclohexene, isobutyraldehyde in 1,2-dichloroethane, and the air was set to 2.6 mL/min and 1920 mL/min, respectively, as used for the above-mentioned continuous operation and no change was made throughout the investigation. The results are displayed in Figure 6. At first, the equivalent of aldehyde was reduced from three to two equivalents and 0.26 M solution of cyclohexene was prepared. As compared to the reaction with a 1:3 ratio of cyclohexene and the aldehyde, the flow epoxidation with a 1:2 ratio of that favorably proceeded to achieve higher levels of yield and productivity (97% yield and 3.9 g/h with a 1:2 ratio of cyclohexene to aldehyde vs 93% yield and 3.3 g/h with 1:3 ratio of cyclohexene and aldehyde). We succeeded in reducing the equivalents of aldehyde without any loss of productivity by our approach. Following is the flow operation with the increased concentration such as 0.52 M and 0.78 M with a 1:2 ratio of cyclohexene and aldehyde. As the concentration increased, the productivity of cyclohexene oxide was amplified up to 11.1 g/h, which is 14-fold that obtained from 0.065 M solution of cyclohexene with 3 equivalents of isobutyraldehyde (0.8 g/h). Consequently, a small change in the equivalents of isobutyraldehyde and concentration of cyclohexene in solution delivered about a 14-fold remarkably large enhancement of productivity. Moreover, using a high concentration solution for flow operation enabled minimization of the volume of reagents used.

## Conclusion

In the present study, we developed the continuous flow system for the aerobic cyclohexene oxide production based on appropriate temperature and residence time control. Moreover, the production of cyclohexene oxide with high yield and selectivity was achieved under the conditions of a very short timescale such as 1.4 min of residence time without added catalyst. Rapid production of cyclohexene oxide in a continuous flow system could be sustained for at least 1 hour with reliable operation stability. In addition, the use of a higher concentration of cyclohexene (1:2 = cyclohexene/isobutyraldehyde in 0.78 M of cyclohexene in solution) results in a remarkable improvement in productivity (11.1 g/h). Further flow condition search will be conducted for developing a highly productive cyclohexene oxide production and other rapid gas-liquid reactions.

## Experimental

### Procedure for epoxidation of cyclohexene with air bubbling in a batch reactor

The same procedure as described in the literature was followed [5]. A 100 ml two-necked flask equipped with a cooling condenser through which −15 °C cooling solvent was circulated, a solution of isobutyraldehyde (9.75 mmol) and tridecane (as an internal standard) in 1,2-dichloroethane (45 mL) was stirred vigorously with air bubbling at the reaction temperature for 30 min to initiate the peroxide formation. Then, a solution of cyclohexene (3.25 mmol) in 1,2-dichloroethane (5 mL) was added. The inner pressure was released through a thin needle on the top of the condenser. The reaction temperature was controlled either in a water bath or an oil bath. At a certain reaction time, 50 μL of the reaction solution was taken out using a gastight syringe and immediately diluted with deuterated chloroform for ^1^H NMR analysis.

### General procedure for epoxidation of cyclohexene with air in the flow microreactor

A flow microreactor system consisting of a T-shaped micromixer, and one microtube reactor was used for epoxidation of cyclohexene with air. A solution of cyclohexene (0.065 M), isobutyraldehyde (0.195 M), and tridecane as an internal standard in 1,2-dichloroethane (flow rate: 2.6 mL/min) exposed to Ar flow was introduced into a mixer by a plunger pump. Air was introduced into the mixer at the flow rate of 480 mL/min. The resulting mixture was passed through the microtube reactor (ϕ = 2.17 mm, *L* = 20 m, residence time = 1.4 min) which was immersed in an oil bath. The reaction mixture was then passed through the microtube ((ϕ = 2.17 mm, *L* = 1 m) immersed in a water bath. The resulting solution was collected in a vessel for 1 min, then a small amount of it was immediately diluted with deuterated chloroform and analyzed by ^1^H NMR to obtain yield and conversion.

## Supporting Information

File 1 Experimental and analytical data.
