# Supplementary material for: Rapid gas–liquid reaction in flow. Continuous synthesis and production of cyclohexene oxide
Source: Beilstein J Org Chem. 2022 Jun 13;18:660–8. doi: 10.3762/bjoc.18.67 (PMC9235905; doi:10.3762/bjoc.18.67)
Supplement: File 1 — Experimental and analytical data. [file Beilstein_J_Org_Chem-18-660-s001.pdf]

BEILSTEIN JOURNAL OF ORGANIC CHEMISTRY

Supporting Information

for

Rapid gas–liquid reaction in flow. Continuous synthesis and production of cyclohexene oxide

Kyoko Mandai, Tetsuya Yamamoto, Hiroki Mandai and Aiichiro Nagaki

Beilstein J. Org. Chem. **2022,** *18,* 660–668. doi:10.3762/bjoc.18.67

Experimental and analytical data

License and Terms: This is a supporting information file under the terms of the Creative Commons Attribution License (https://creativecommons.org/licenses/by/4.0). Please note that the reuse, redistribution and reproduction in particular requires that the author(s) and source are credited and that individual graphics may be subject to special legal provisions.
The license is subject to the *Beilstein Journal of Organic Chemistry* terms and conditions: (https://www.beilstein-journals.org/bjoc/terms)

Table of Contents

General	S2
Experimental	S2
General procedure for epoxidation of cyclohexene with air bubbling in batch reactors	S2
General procedure for epoxidation of cyclohexene with air in flow reactors	S2
**Table S1.** Yield of cyclohexene oxide in epoxidation of cyclohexene with air bubbling in batch at various temperature.	S3
**Table S2.** Investigation of reaction temperature in flow epoxidation of cyclohexene at residence time of 0.35 min.	S3
**Table S3.** Investigation of residence time in flow epoxidation of cyclohexene at 100 °C	S3
**Table S4.** Continuous production of cyclohexene oxide	S4
**Table S5.** Effect of concentration of cyclohexene and equivalent of aldehyde.	S4
**Figure S1.** (a) ^{1}H NMR spectra in $CDCl_3$ of the solution of cyclohexene and isobutyraldehyde in 1,2-dichloroethane (b) ^{1}H NMR spectra in $CDCl_3$ of the eluted solution collected in the 1-hour continuous flow experiment (>99% conv, 91% yield)	S5
**Figure S2.** (a) ^{1}H NMR spectra in $CDCl_3$ of the eluted solution (flow conditions: residence time; 3 min, at 120 °C, 98% conversion, 15% yield) after drying with heating under reduced pressure. (b) ^{1}H NMR spectra in $CDCl_3$ of poly(cyclohexene oxide.	S6
**References**	S6

General

GC-MS analysis was performed on SHIMADZU GCMS-QP2020NX using a fused silica capillary column (Agilent, HP-PONA; 0.20 mm × 50 m; initial oven temperature, 40 °C). ^{1}H and ^{13}C NMR spectra were recorded on a JEOL JNM-ECZ400S (^{1}H 400 MHz, ^{13}C 100 MHz) spectrometer with Me_4Si or $CDCl_3$ as a standard in $CDCl_3$ unless otherwise noted. Cyclohexene, isobutyraldehyde, and tridecane were purchased from Tokyo Chemical Industry Co., Ltd. and used without further purification. 1,2-dichloroethane was purchased from Fujifilm Wako Pure Chemical Corporation and used without further purification. The air cylinder was purchased from Medical Sakai

Stainless steel (SUS316) microtube reactors with inner diameter of 1.00 and 2.17 mm were purchased from GL Sciences and were cut or connected into appropriate lengths The micromixer and microtube reactors were connected with stainless steel fittings (GL Sciences) to construct the flow microreactor in the laboratory. The flow microreactor was dipped in an oil bath to control the temperature. Solutions were continuously introduced to the flow microreactor using a plunger pump, DFC FC-F-PP-110S.

Experimental.

General procedure for epoxidation of cyclohexene with air bubbling in batch reactors

The same procedure as described in the literature was followed [1]. In a 100 ml two-necked flask equipped with a cooling condenser through which −15 °C cooling solvent was circulated, a solution of isobutyraldehyde (9.75 mmol) and tridecane as an internal standard in 1,2-dichloroethane (45 mL) was stirred vigorously with air bubbling at reaction temperature for 30 min. Then, a solution of cyclohexene (3.25 mmol) in 1,2-dichloroethane (5 mL) was added. Inner pressure was released from a thin needle on the top of the condenser. The reaction temperature was controlled either in a water bath or an oil bath. At a certain reaction time, 50 μL of the reaction solution was taken out using a gastight syringe and immediately diluted with deuterated chloroform for ^{1}H NMR analysis to determine the conversion of cyclohexene and yield of cyclohexene oxide.

General procedure for epoxidation of cyclohexene with air in flow reactors

A flow microreactor system consisting of a T-shaped micromixer and one microtube reactor was used for the epoxidation of cyclohexene with air. A solution of cyclohexene (0.065 M), isobutyraldehyde (3 equivalents, 0.195 M), and tridecane as an internal standard in 1,2-dichloroethane (flow rate: 2.6 ml/min) exposed to Ar flow was introduced into a mixer by a plunger pump. Air was introduced into a mixer at the flow rate of 480 ml/min. The resulting mixture was passed through the microtube reactor (ϕ = 2.17 mm, L = 20 m, residence time = 1.4 min) which was immersed in an oil bath. The reaction mixture was then passed through the microtube (ϕ = 2.17 mm, L = 1 m) immersed in a water bath. The resulting solution was collected in a vessel for 1 min, then a small amount of it was immediately diluted with deuterated chloroform and analyzed by ^{1}H NMR to obtain the conversion of cyclohexene and yield of cyclohexene oxide.

Data table

Table S1. Yield of cyclohexene oxide in the epoxidation of cyclohexene with air bubbling in batch at various temperatures.

	reaction time (min)					
temperature	30	60	120	180	270	570
40 ^{o}C	11%	15%	17%	19%	-	-
60 ^{o}C	32%	42%	62%%	72%	75%	74%
80 ^{o}C	17%	24%	30	41%	-	-

Yield of cyclohexene oxide was determined by ^{1}H NMR analysis.

Table S2. Investigation of the reaction temperature in flow epoxidation of cyclohexene at a residence time of 0.35 min.

temperature (^{o}C)	conversion (%)[a]	yield (%)[a]	yield/conversion (%)
40	19	0	
60	28	1	5
80	41	12	28
100	73	47	64
120	90	47	52

[a] Determined by ^{1}H NMR analysis.

Table S3. Investigation of residence time in flow epoxidation of cyclohexene at 100 ^{o}C.

residence time（min)	conversion (%)[a]	yield (%)[a]
0.14	43	26
0.35	73	47
0.7	90	70
1.4	98	84
2	100	46

[a] Determined by ^{1}H NMR analysis.

Table S4. Continuous production of cyclohexene oxide

duration time (min)	conversion (%)[a]	yield (%)[a]	integrated productivity (g/h)[b]
0-5	100	94	0.3
5-10	100	91	0.6
10-15	100	94	0.9
15-20	100	91	1.2
20-25	100	93	1.5
25-30	100	92	1.8
30-35	100	93	2.1
35-40	100	91	2.4
40-45	100	92	2.8
45-50	100	91	3.1
50-55	100	93	3.4
55-60	100	94	3.7

[a] Determined by ^{1}H NMR analysis. [b] Calculated based on theoretical productivity for 5 min operation and yield of cyclohexene oxide.

Table S5. Effect of concentration of cyclohexene and equivalent of aldehyde.

concentration of cyclohexene (M)[a]	ratio of cyclohexene and aldehyde (cyclohexene : aldehyde)	yield (%)[b]	productivity (g/h)[c]
0.065	1 : 3	84	0.8
0.26		93	3.3
0.26	1 : 2	97	3.9
0.52		95	7.6
0.78		93	11.1

[a] Solution in 1,2-dichloroethane with isobutyraldehyde and tridecane. [b] Determined by ^{1}H NMR analysis. [c] Calculated based on theoretical productivity and yield of cyclohexene oxide.

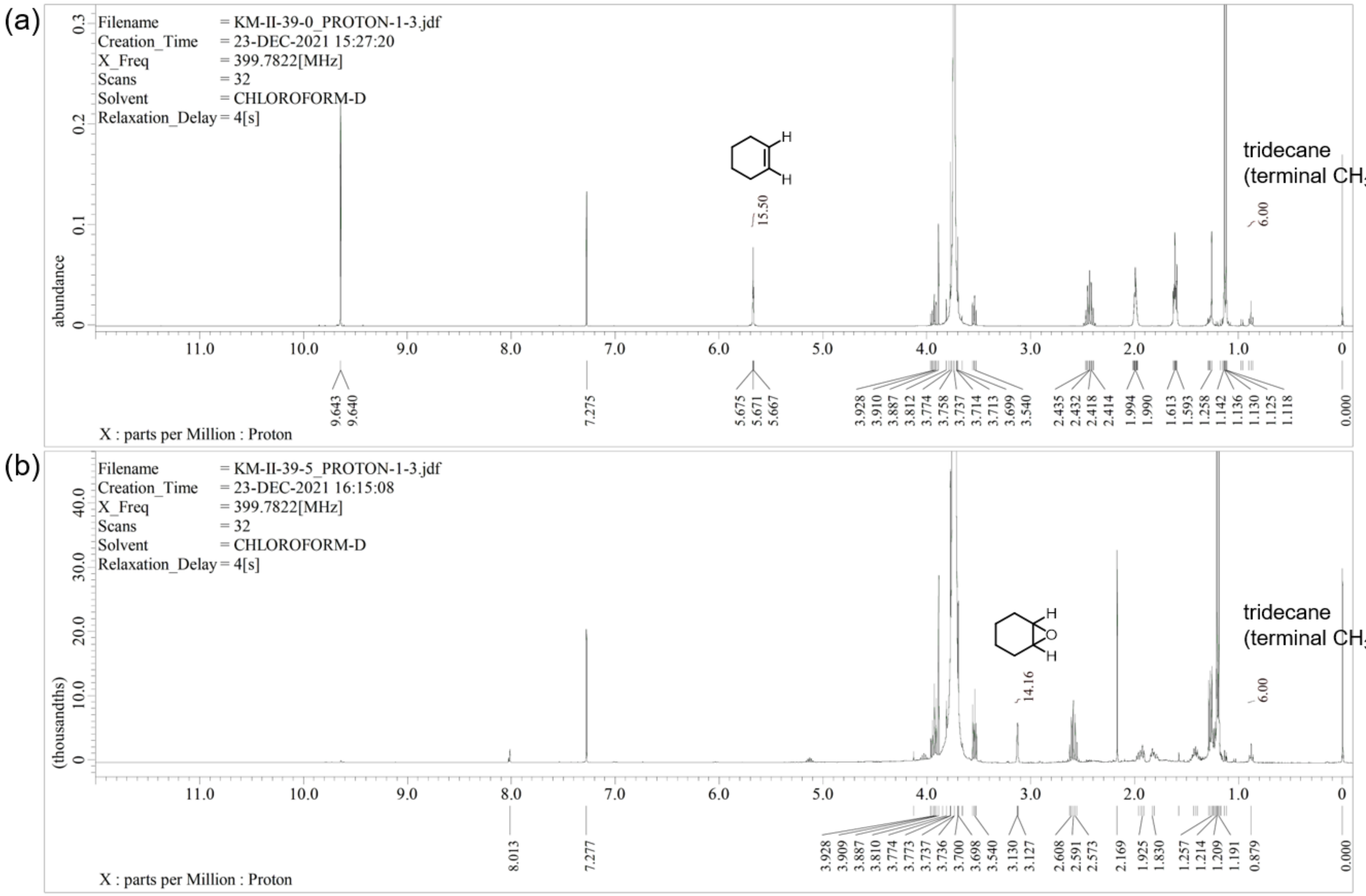

Figure S1. (a) ^{1}H NMR spectra in $CDCl_3$ of the solution of cyclohexene and isobutyraldehyde in 1,2-dichloroethane (b) ^{1}H NMR spectra in $CDCl_3$ of the eluted solution collected in the 1-hour continuous flow experiment (>99% conv, 91% yield)

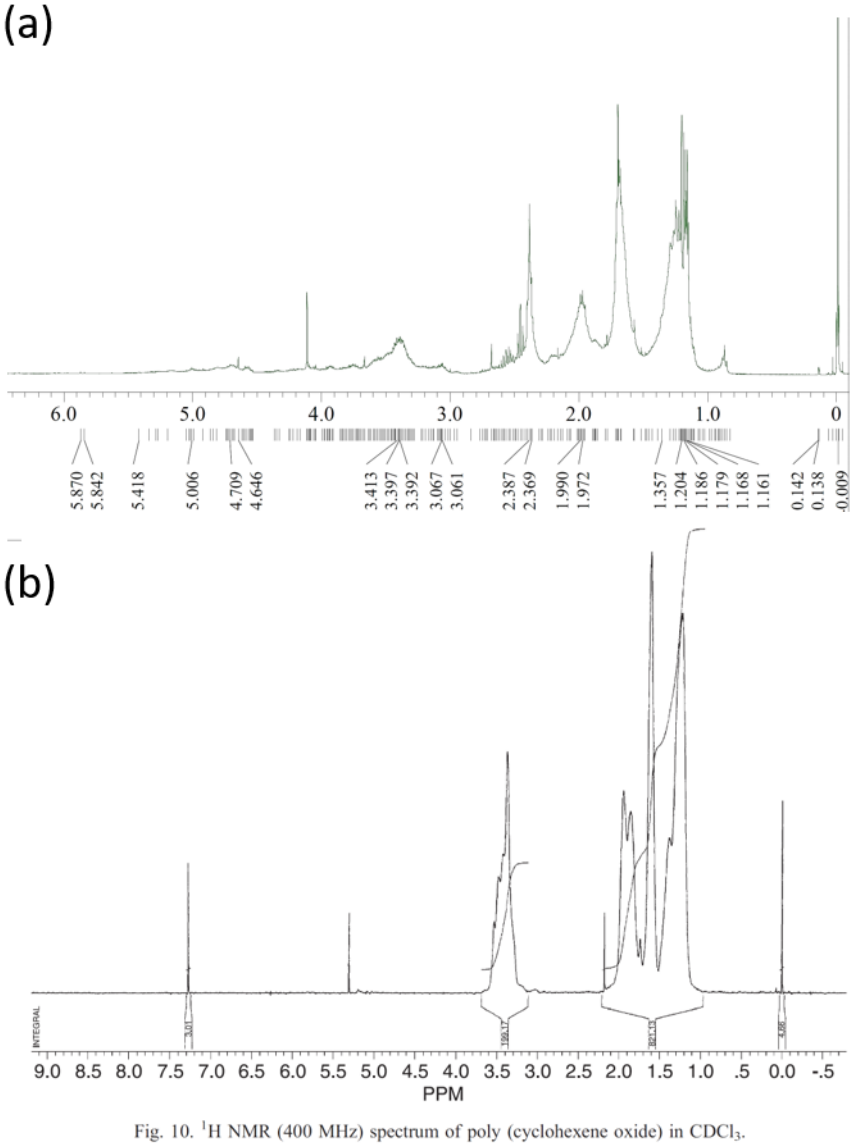

Figure S2. (a) ^{1}H NMR spectra in $CDCl_3$ of the eluted solution (flow conditions: residence time; 2 min, at 120 ^{o}C, 98% conversion, 15% yield) after drying with heating under reduced pressure. (b) ^{1}H NMR spectra in $CDCl_3$ of poly(cyclohexene oxide reported in [2].

References

1. Kaneda, K.; Haruna, S.; Imanaka, T.; Hamamoto, M.; Nishiyama, Y.; Ishii, Y. *Tetrahedron Lett.* **1992**, *33*, 6827–6830.
2. Yahiaoui, A.; Belbachir, M.; Soutif, J. C.; Fontaine, L. *Mater. Lett.* **2005**, *59*, 759–767.
